# Gradient Microstructure Design in Stainless Steel: A Strategy for Uniting Strength-Ductility Synergy and Corrosion Resistance

**DOI:** 10.3390/nano11092356

**Published:** 2021-09-10

**Authors:** Qiong He, Wei Wei, Ming-Sai Wang, Feng-Jiao Guo, Yu Zhai, Yan-Fei Wang, Chong-Xiang Huang

**Affiliations:** 1 School of Aeronautics and Astronautics, Sichuan University, Chengdu 610065, China; qionghescu@163.com (Q.H.); double_w@foxmail.com (W.W.); mingsaiwangscu@163.com (M.-S.W.); guofengjiaoscu@163.com (F.-J.G.); yuzhai2021scu@163.com (Y.Z.); 2 Department of Mechanics and Engineering Science, College of Engineering, Peking University, Beijing 100871, China

**Keywords:** stainless steel, heterostructure, strength and ductility, corrosion resistance, phase reversion

## Abstract

Martensite transformation and grain refinement can make austenitic stainless steel stronger, but this comes at a dramatic loss of both ductility and corrosion resistance. Here we report a novel gradient structure in 301 stainless steel sheets, which enables an unprecedented combination of high strength, improved ductility and good corrosion resistance. After producing inter-layer microstructure gradient by surface mechanical attrition treatment, the sheet was annealed at high temperature for a short duration, during which partial reverse transformation occurred to form recrystallized austenitic nano-grains in the surface layer, i.e., introducing extra intra-layer heterogeneity. Such 3D microstructure heterogeneity activates inter-layer and inter-phase interactions during deformation, thereby producing back stress for high yield strength and hetero-deformation induced (HDI) hardening for high ductility. Importantly, the recrystallized austenitic nano-grains significantly ameliorates the corrosion resistance. These findings suggest an effective route for evading the strength–ductility and strength–corrosion tradeoffs in stainless steels simultaneously.

## 1. Introduction

Austenitic stainless steels are the most common workhorse material for structural applications in corrosive circumstances [1,2,3]. However, in practical engineering, the available stainless steels are generally composed of homogeneous austenitic coarse-grains (CG) and display limited yield strength. For example, the yield strength of hot forged commercial 301 stainless steel is only about 205–380 MPa, which is far weaker than the high-strength steels strengthened by precipitates and/or heterogeneous phases [4]. Therefore, to minimize material cost and improve engineering safety in extreme service conditions, advanced high-strength stainless steels with promising ductility and corrosion resistance are highly desirable. This presents a new challenge for material developers.

Refining the grains and phases to ultrafine size or even nanoscale can improve the strength to GPa level, but this usually comes at a dramatic loss of ductility [5]. Recent advances in microstructure design frequently found that this tradeoff can be largely overcome by purposely deploying trans-scale heterogeneous microstructure, such as designing multimodal [6,7], harmonic [8,9], gradient [10,11,12,13,14], laminate and lamella structures [15,16,17,18,19]. Among various heterostructures, the gradient structures, which consist of nanostructured (NS) surface layers sandwiching coarse-grains interior, exhibit the greatest potential for engineering application because gradient microstructure in bulk with various shapes can be readily synthesized by surface plastic deformation techniques [20,21]. In addition to combining the intrinsic superiority of heterogeneous layers, such as the high strength of nanostructured surface and high ductility of the coarse-grains interior, the mechanical incompatibility-induced inter-layer interaction can contribute to extra strengthening and work hardening by awakening long-range internal stresses [15,18,22,23,24]. Unprecedented strength–ductility synergy superior to the predictions of volume fraction-weighted rule of the mixture was indeed achieved in several gradient-structured materials [10,11,12,25,26]. These advances suggest the possibility of optimizing the mechanical performances of stainless steels by designing gradient microstructure.

For stainless steel, a nanostructured surface layer processed by severe plastic deformation should contain dense dislocations and a large fraction of nano-martensite due to the activation of transformation-induced plasticity (TRIP) and defects accumulation [3,27,28,29,30,31]. Electrochemical corrosion tests revealed that nanostructured provide more nucleation sites, adhesion work and homogeneous capillary force to improve the compactness of passive film [1,32,33,34]. Such a positive passivation effect is favored for improving corrosion resistance. However, a passive film cannot stably adhere to nano-martensite, which leads to the formation of a defective passive film, thereby accelerating corrosion damage [34,35]. The beneficial effect of nanostructured may be completely nullified if the martensite content is large enough [33,34]. This raises a critical question: while maintaining the nano-sized microstructure for high strength, how does one tune the martensite content in surface layers to improve the corrosion resistance? 

Inspired by the above challenges, here we provide a new strategy that can produce an ideal gradient structure in stainless steel: controlled annealing to activate partial reverse transformation in the nanostructured surface layer of gradient structure. Systematical tests suggest that an optimized combination of mechanical properties and corrosion resistance are indeed achievable. 

## 2. Experiments and Gradient Structure Preparation

**Figure 1:**
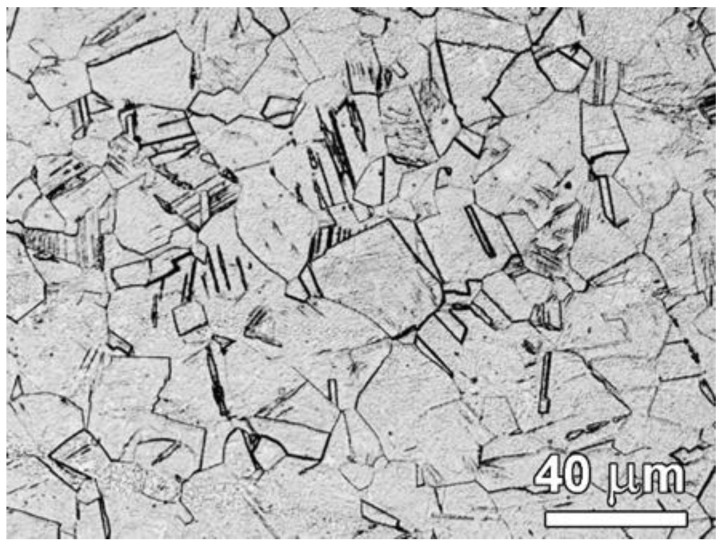
Optical image showing the coarse-grains microstructure of raw material.

A 1-mm-thick 301 stainless steel plate was selected as the raw material, which has a chemical composition of 16.82 Cr, 7.41 Ni, 1.43 Mn, 0.73 Si, 0.098 C, 0.017 P, 0.007 S and the balance of Fe (all in mass.%). The as-received material is composed of homogenous austenitic coarse-grains (Figure 1). The samples for optical observation were first ground with silicon carbide (SiC) papers down to 4000 grit and then polished with diamond pastes (6, 3 and 1 μm). Moreover, the samples were etched by 2 mL HF + 3 mL HNO_3_ + 95 mL H_2_O at room temperature for 20 s and observed by an optical microscope (OM) with a BX53M-UC6.0 microscope (OLYMPUS, Beijing, China). The surface mechanical attribution treatment (SMAT) technique was used to produce the gradient structured 301ss specimens, with a central coarse-grained layer sandwiched between two gradient layers. The treatment process is mainly to perform a large amount of shot peening on the surface of the sample in a short time through stainless steel balls (shots) placed at the bottom of a cylinder-shaped vacuum chamber. During the SMAT process, spherical steel shots of 3-mm in diameter were accelerated to high speeds using high-power ultra-sound to impact the sample disk. The impact speed is 20 m/s. Both sides were symmetrically treated for 10 min. Thereafter, three sets of as-SMATed samples were annealed at 900 °C for 5 s, 10 s and 20 s, respectively. For convenience, the gradient structures subjected to further annealing are labeled as SA*_x_*_s_ samples, where the subscript (*x*s) represents the specific annealing time. For details on the selection of SMAT processing time and annealing parameters, please refer to Figures S1–S3.

Dog-bone-shaped tensile specimens with a gauge dimension of 12 × 2 × 1 mm^3^ were machined from as-processed gradient plates. Uniaxial tensile tests were carried out at room temperature at a strain rate of 5 × 10^−4^ s^−1^ under a SHIMADZU AG-100KN experimental machine. Vickers microhardness was achieved using Vickers MHV-705 hardness tester equipment (Baoling, Shanghai, China). Mechanical polishing to 2000# to remove the surface stress layer. The Vickers hardness test load was 50 g, and the retention time was 15 s. The Vickers hardness indentation along the gradient direction of the sample structure is arranged in a “Z” shape with the indentation spacing greater than 3 times the diagonal length. In order to minimize test error or characterize structural inhomogeneity within the layer, the hardness of each model sample is repeatedly tested at 3 to 15 independent locations.

Transmission electron microscope (TEM) was performed under an FEI Talos F200X microscope (Thermo Fisher Scientific, Shanghai, China), and X-ray diffraction (XRD) was conducted in an X’ Pert Pro MPD DY129 equipment with diffracted beam monochromator. The microstructure was observed using a TEM at 200 kV. The samples for TEM were prepared by mechanically polishing to a thickness of ~50 μm and then electronically polished at −10 °C by a twin-jet. A focused ion beam was used to prepare TEM foils from designated layers. XRD was utilized to examine the phase composition at different depths. For the purpose of conducting independent measurements at designated depths, the sample was prepared by polishing away the redundant surface layers. XRD measurements were performed using Cu*Kα* radiation (lambda = 0.15418 nm) within the scanning range of 40–90° at 0.02° s^−1^. The volume fraction of phases was evaluated from the integrated intensity of diffraction peaks (I_hkl_) after background subtraction. The raw XRD data were processed by Rietveld refinement using the TOPAS tool to analyze the phase content.

The specimens for electrochemical tests were machined as 10 mm × 10 mm slices. Electrochemical potentiodynamic polarization tests were carried out in a 3.5 wt.% NaCl solution at 25 °C. Stainless steel plays an irreplaceable role in the process of human exploration of the ocean. Therefore, 3.5 wt.% NaCl solution was often chosen in electrochemical tests to simulate the marine environment to study the effect of seawater on the corrosion resistance of stainless steel. The electrical conductivity of the solution was less than 2 × 10^−6^ S/cm. The reference electrode was a saturated calomel electrode, and a platinum electrode was used as the auxiliary one.

## 3. Results and Discussion

### 3.1. Gradient Structures

**Figure 2:**
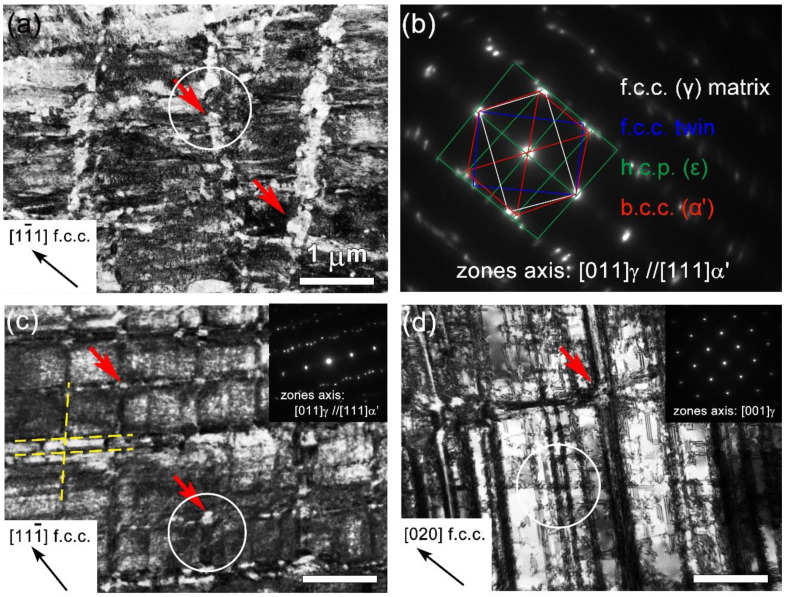
Representative TEM images showing the microstructure gradient of as-SMATed sample: (**a**) at a depth of about 50 μm from the surface, (**b**) the corresponding SAED pattern of microstructure in (**a**,**c**) about 100-μm-deep and (**d**) about 250-μm-deep. The red arrows mark the granular martensite, and the dotted yellow lines in (**c**) indicate the martensitic nano-lamellae. (**a**,c,**d**) share the same ruler, and the white circle is selected as the SAED test area.

Figure 2 presents the representative microstructure at varying depths of the as-SMATed gradient sample. In Figure S4, austenite is the matrix, twins and α’-martensite are marked with blue and red arrows respectively, and ε-martensite is the green dashed part. As shown (Figure 2a), the layer at a depth of ~50 μm is composed of significantly refined nanostructured with an extremely high density of dislocations. Granular α’-martensite in size of a few tens or hundreds of nanometers are frequently observed, as partially indicated by red arrows. The selected area electron diffraction pattern (SAED, Figure 2b) confirms that this layer is a complex composite containing γ austenite matrix, ε-martensite, α’-martensite and twins. The ε-martensite is the intermediate phase of martensite transformation [36]. This indicates severe microstructure subdivision, transformation-induced plasticity and twinning-induced plasticity experienced by the surface layers.

At a depth of ~100 μm, the austenite matrix is subdivided by intersecting martensitic nano-lamellae, forming discrete quadrilateral islands in the size of submicron (Figure 2c). Except for relatively weak martensitic spots, there is no dramatic change in the SAED pattern (the insert) compared to that of the surface layer (Figure 2b). As the depth increased to ~250 μm (Figure 2d), the microstructure is obviously coarsened, and the densities of dislocations and granular martensite are reduced. As shown, the plastic deformation at this depth is mainly accommodated by long parallelepiped bands, which rarely intersect with each other, suggesting reduced accumulation of plastic strain. 

**Figure 3:**
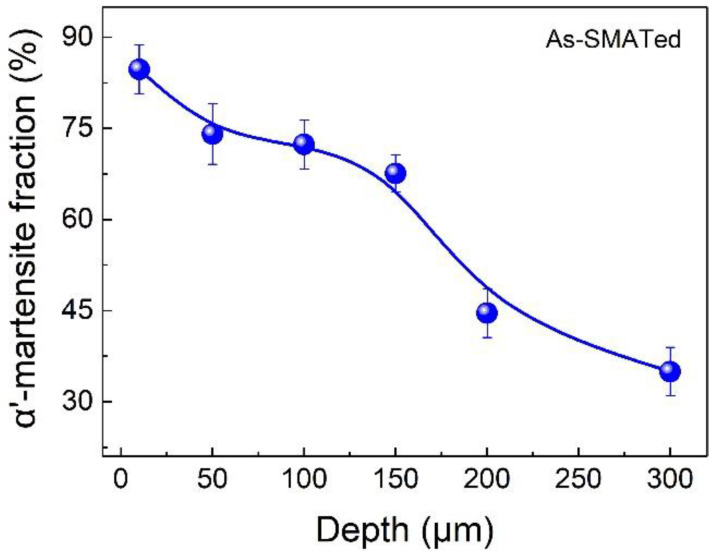
Variation of α’-martensite volume fraction along the depth.

We statistically analyzed the change of phase content along depth basing on XRD measurements (Figure 3). The volume fraction of martensite at a depth of ~10 μm is as high as ~84%, implying that most γ-austenite has been transformed into martensite by SMAT treatment. With increasing depth, martensite content decreases gradually. For the ~300-μm-deep layer, it is decreased to only about 37%.

**Figure 4:**
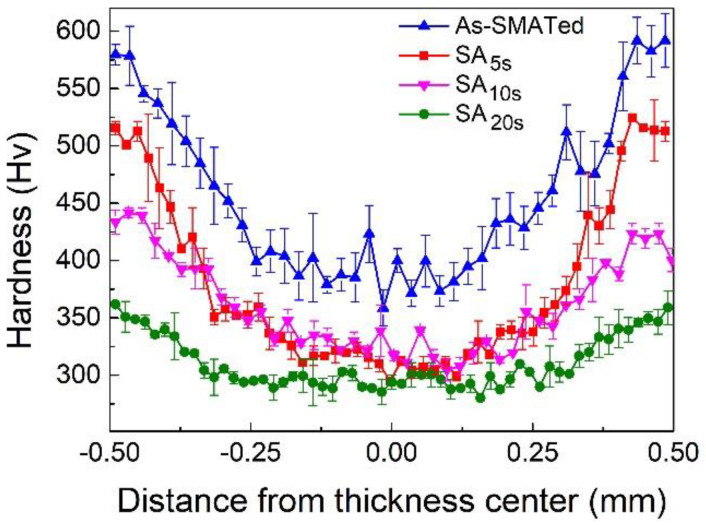
Cross-sectional hardness distribution of as-SMATed and SA*_x_*_s_ gradient samples.

These results suggest that the microstructure gradient along depth exists in grain size, phase content and dislocation density. The formation of microstructure gradient is primarily due to the gradients in accumulated plastic strain and strain rate during SMAT treatment, which decays exponentially from surface to interior [37,38,39]. As the cross-sectional hardness profile shown in Figure 4 (the blue data), the hardness gap between the nanostructured surface layer and the coarse-grains interior is as large as ~210 Hv, and the total thickness of layers with mechanical gradient is ~350 μm. Such a large hardness difference is expected to allow for significant deformation incompatibility between layers [11,25,26,40]. 

After annealing at 900 °C for a short duration, there is an obvious recovery in hardness, especially in the nanostructured surface layers (the data of SA*_x_*_s_ samples in Figure 4). However, the hardness difference from nanostructured surface to the coarse-grains interior in SA_5s_ and SA_10s_ samples is still as large as ~200 Hv and ~140 Hv, respectively, indicating that considerable mechanical and microstructure gradients are retained. 

### 3.2. Improved Mechanical Responses and Corrosion Resistance

**Figure 5:**
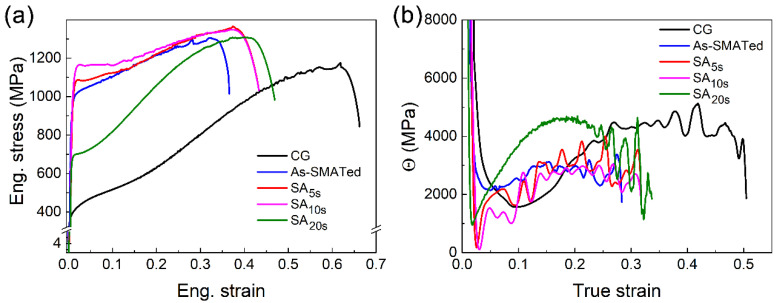
Comparison of the tensile responses of gradient samples: (**a**) engineering stress-strain curves and (**b**) strain hardening rate (Θ).

The tensile responses of the coarse-grains sample, as-SMATed and SA*_x_*_s_ gradient structures, are presented in Figure 5. Three interesting points can be drawn from the comparison among them. First, the as-SMATed gradient structure indeed exhibits an acceptable combination of strength and ductility, with a yield strength of ~1000 MPa and uniform elongation of ~30%. This can be attributed to (i) the hetero-deformation induced strengthening and hardening that is accompanied by strain gradient accumulation and back stress development [18,23,41], and (ii) the strong transformation-induced plasticity effect enhanced by gradient microstructure [3,27]. 

Second, the yield strength of SA_5s_ and SA_10s_ samples are further enhanced compared to that of the as-SMATed gradient structure (see the red and pink data). As evidenced by the hardness profile (Figure 4), annealing causes serious softening, which should result in a dramatic drop in tensile yield strength according to our common sense. However, the yield strength of the SA_10s_ sample (~1170 MPa) is actually higher than both that of SA_5s_ (~1090 MPa) and as-SMATed (~980 MPa) samples. More surprisingly, such extra strength comes with no consumption of ductility, and on the contrary, the uniform elongation even increased by ~5%, leading to more superior strength–ductility synergy. This annealing-induced strengthening is contrary to our general knowledge from textbooks. Reasoning arising therefrom, therefore, is that a unique microstructure should be formed during short-term annealing, and it enables extremely efficient extra strengthening under tension.

Third, the SA*_x_*_s_ gradient structures retain high work hardening capability (Figure 5b). After short-time annealing, the strain hardening rate (Θ) is recovered significantly within the strain range of ~0.1–0.2, resulting in an obvious Θ up-turn due to transformation-induced plasticity effect like in the coarse-grains sample. 

**Figure 6:**
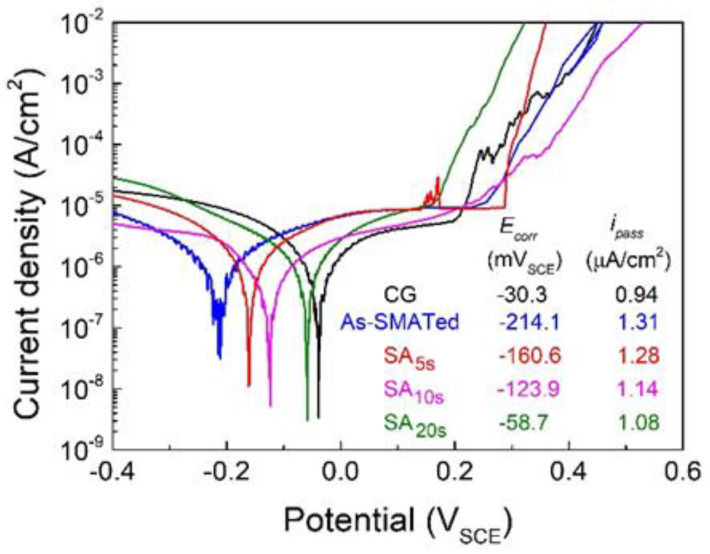
Potentiodynamic polarization curves of as-SMATed and SA*_x_*_s_ gradient samples. The curve of homogeneous coarse-grains is also plotted for comparison. The value of *E_corr_* and *i_pass_* are averaged from three independent tests.

A comparison of potentiodynamic polarization behavior in the 3.5 wt.% NaCl solution among homogeneous coarse-grains, as-SMATed and SA*_x_*_s_ gradient structures is shown in Figure 6. The corrosion potential (*E_corr_*) and average passive current density (*i_pass_*) are obtained from the polarization curves by Tafel extrapolation (insert in Figure 6). For the as-SMATed gradient structure, the *E_corr_* is much lower, and the *i_pass_* is higher than that of homogeneous coarse-grains, suggesting a strong deleterious effect of SMAT treatment on the corrosion resistance of 301 stainless steel. This may be attributed to the large-fraction nano-martensite and high-density crystallographic defects accumulated in the nanostructured surface layer (Figure 2a). It has been widely examined that a large volume fraction of martensite renders a galvanic effect between austenite and martensite phases, which causes a cathodic shift in *E_corr_* and thus decreases the corrosion resistance significantly [34,35,42,43]. Dislocations and other defects with high-distortion energy can reduce the electron work function and the energy barrier for electrochemical reactions [44]. High-density defects, therefore, can provide more active sites to increase the corrosion rate [45]. Such reduced corrosion resistance is one of the key reasons why the conventional gradient-structured stainless steel is not widely welcomed in practical engineering, although it indeed enables an acceptable strength–ductility synergy (Figure 5a). 

Interestingly, for the gradient structures subjected to further annealing treatment, the *E_corr_* increases and the *i_pass_* decreases gradually with increasing annealing time. The SA_10s_ sample exhibits a moderate corrosion resistance much better than the as-SMATed structure. When the annealing time is increased to 20 s, i.e., for the SA_20s_ gradient structure, the yield strength is still twice that of the homogeneous coarse-grains (Figure 5a), but their corrosion resistance is almost comparable (Figure 6). These results suggest that the unique microstructure formed by annealing is effective in enhancing corrosion resistance as well.

### 3.3. Physics behind the Mechanical Responses and Corrosion Resistance

**Figure 7:**
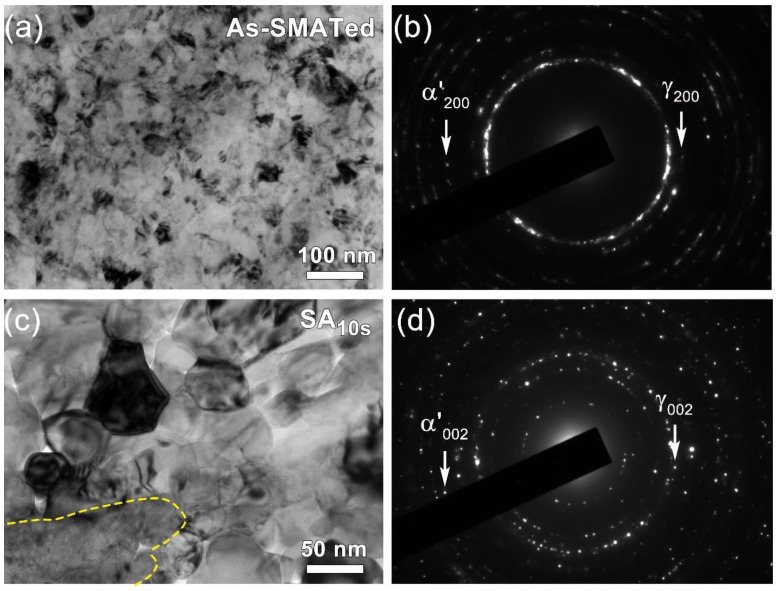
TEM images taken from a depth of ~10 μm in (**a**,**b**) as-SMATed gradient sample and (**c**,**d**) SA_10s_ sample, showing the partial reverse transformation and recrystallized austenitic nano-grains in the latter. Comparing the SAED patterns in (**b**,**d**) suggests dramatic enhancement of austenitic spots after annealing.

Here, the remaining question is what kind of microstructure after short-time annealing results in improved strength, enhanced work hardening and good corrosion resistance simultaneously. To answer this question, we take the SA_10s_ gradient structure as an example to examine the microstructure change in the topmost surface layer (Figure 7). As the typical TEM image and the corresponding SAED pattern presented in Figure 7c,d, the high-temperature annealing renders quick reverse transformation from the high-distortion zone to nucleate new austenitic grains. Interestingly, the extremely short annealing duration (5–10 s) enables two critical effects: (i) the recrystallized grains have no chance to grow up, forming equiaxed nano-grains in diameter of only tens or a few hundred nanometers, and (ii) the reverse transformation cannot evolve completely, leaving un-recrystallized islands (the domain marked by yellow cycle) embedded in austenite domains. In contrast to the martensite-dominated homogeneous nanostructured layer in the topmost of the as-SMATed sample (Figure 7a,b), the partial reverse transformation results in significant intra-layer heterogeneity, with a dramatic difference in phase attributes, dislocations density and grain size between recrystallized and un-recrystallized domains. Such extra microstructure heterogeneity makes the sample a 3D heterostructure.

Under the corrosive circumstance, the recrystallized nanocrystalline materials with dense grain boundary but low distortion is favored by the diffusion of Cr, which provides more nucleation sites and adhesion work to form compact passive film [1,32]. On the other hand, recrystallization reduces the dislocation density and martensite content in surface layers, which improves the corrosion resistance by reducing the active sites and increasing the energy barrier of electrochemical reaction [44,45]. These are the reasons why corrosion resistance recovered dramatically after short-time annealing, and the SA_10s_ gradient sample achieved a moderate corrosion resistance (Figure 6).

Importantly, the unique 3D heterostructure is effective in improving strength. First, the recrystallized austenitic grains remain extremely small in size, which enables high grain boundary strengthening. Second, in addition to the back stress enabled by a cross-layer accumulation of geometrically necessary dislocations (GNDs) [15,23,24,25,46], the intra-layer microstructure inhomogeneity should contribute to extra back stress strengthening as well. The yielding of heterostructure is a process that plastic events are activated progressively in heterogeneous domains [11]. For the nanostructured surface layer in SA*_x_*_s_ samples, dislocation activity should be activated firstly in the recrystallized austenitic domains during yielding. However, the early dislocations cannot glide through the domain boundary since the neighboring martensitic domain still deforms elastically [23,47]. This leads to the formation of GNDs pile-up against domain boundary in order to accommodate the plastic strain incompatibility across it [48]. The GNDs pile-up exerts long-range back stress to repel more dislocation emission in the austenitic domain, i.e., an effect of offsetting the resolved effective stress, thereby making the austenitic domain appear stronger [23]. As measured in hetero-lamella Ti and coarse-grains/nanostructured laminate [15,18], the global back stress can be as high as >60% of the yield strength, as long as the domain interface is strong enough. These suggest that the extra back stress arising from intra-layer heterogeneity may be the key physics behind the high yield strength of the SA_10s_ sample (Figure 5a).

At the plastic strain stage, the incompatibility in plastic stability between coarse-grains interior and nanostructured surface rises inter-layer constraint, and the strain partitioning between the recrystallized austenitic domain and the remained martensitic domain in nanostructured surface layer can activate intra-domain constraint [25,49,50]. These constraints lead to further accumulation of 3D hierarchical strain gradient, thereby promoting the development of both back stress in the softer layer/domain and forward stress in the harder layer/domain, and consequently causing extra hetero-deformation induced (HDI) hardening [23,41]. Moreover, the recrystallized austenitic domains in the nanostructured surface layer are intrinsically high in work hardening capability. The high long-range internal stress can, in turn, enhance the transformation-induced plasticity effect [3,27]. These physics are responsible for the high strain hardening rate and good ductility of SA*_x_*_s_ samples (Figure 5b). 

**Figure 8:**
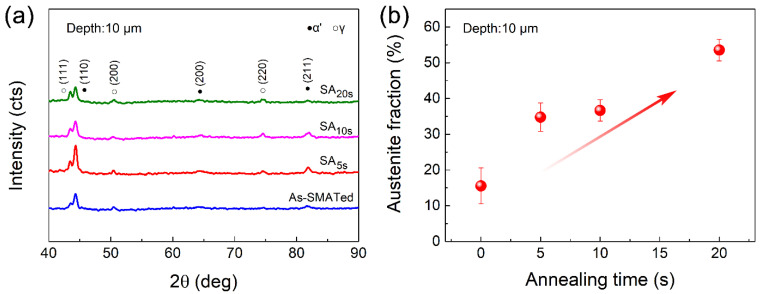
(**a**) XRD patterns obtained in the topmost surface layer of gradient samples after partial reverse transformation annealing. (**b**) The increase of austenite in the topmost surface layer with increasing annealing time.

The fraction of reverse transformation increases with increasing annealing time (Figure 8). When the annealing time is too long, the intra-layer heterogeneity of the nanostructured surface layer would be reduced due to the decreased density of the hetero-domain interface [17,22]. As a result, the heterogeneity-dependent back stress strengthening at yielding will also be reduced. This is also the key reason for the reduced yield strength of the SA_20s_ sample besides the coarser microstructure (Figure 5a). Interestingly, this may suggest the existence of a critical annealing time, for which the intra-layer heterogeneity enables the maximum extra back stress strengthening. Another interesting result is that the yield strength improvement of the SA_10s_ sample is accompanied by a hardness reduction, as compared to that of an as-SMATed structure (Figure 4 and Figure 5a). A suspect is that the effects of heterogeneity-induced back stress can be largely shielded if the strain is extremely severe, such as under indentation [23]; these advocates more in-depth investigations. 

## 4. Conclusions

In summary, here we provide a new answer for the challenge: how to evade the strength–ductility and strength–corrosion tradeoffs simultaneously in stainless steel? The main conclusions are:

(i) The challenge can be overcome by designing gradient microstructure with partial austenitic nano-grains in the surface layer. Such gradient structure can be synthesized by a two-step route: conducting surface severe plastic deformation firstly to produce gradient structure, followed by high-temperature but short-time annealing to active partial reverse transformation in the surface layer.
(ii) The as-processed gradient material is a 3D heterostructure with extra intra-layer heterogeneity in the surface layer, which produces extra back stress for high strength and extra HDI hardening for high ductility. The nano-sized microstructure with reduced dislocation density and martensite content in the surface layer is primarily responsible for the improved corrosion resistance.
(iii) Two advanced samples in 301 stainless steel are provided. The SA_10s_ sample has a high yield strength of ~1200 MPa, a uniform elongation of ~32% and moderate corrosion resistance, which is suitable for high-strength applications. The SA_20s_ sample has superior corrosion resistance and a moderate yield strength of ~700 MPa, which can be served in highly corrosive circumstances.

## Data Availability

The data presented in this study are available on request from the corresponding author.

## Supplementary Materials

The following are available online at https://www.mdpi.com/article/10.3390/nano11092356/s1, Figure S1: (a) Cross-sectional hardness distribution. (b) Variation of martensite volume fraction along depth. Three types of gradient plates were symmetrically treated on both sides for 1 min, 5 min and 10 min, which are labeled as S1min, S5min and S10min samples, respectively, Figure S2: The surface hardness of As-SMATed samples as a function of annealing temperature, Figure S3: The surface hardness of As-SMATed samples as a function of annealing time, Figure S4: TEM image showing the austenite, twins, ε-martensite and α’-martensite of as-SMATed sample.
