# Supplementary material for: Gradient Microstructure Design in Stainless Steel: A Strategy for Uniting Strength-Ductility Synergy and Corrosion Resistance
Source: Nanomaterials (Basel). 2021 Sep 10;11(9):2356. doi: 10.3390/nano11092356 (PMC8472316; doi:10.3390/nano11092356)
Supplement: Supplementary file 1 [file nanomaterials-11-02356-s001.zip › nanomaterials-1358575-SI-conversion.pdf]

nanomaterials

MDPI

Supplementary Materials

Gradient Microstructure Design in Stainless Steel: A Strategy for Uniting Strength–Ductility Synergy and Corrosion Resistance

Qiong He [1], Wei Wei [1], Ming-Sai Wang [1], Feng-Jiao Guo [1], Yu Zhai [1], Yan-Fei Wang [2,]* and Chong-Xiang Huang [1,]*

[1] School of Aeronautics and Astronautics, Sichuan University, Chengdu 610065, China; qionghescu@163.com (Q. H); double_w@foxmail.com (W. W); mingsaiwangscu@163.com (M.S. W); guofengjiaoscu@163.com (F.J. G); yuzhai2021scu@163.com (Y. Z)

[2] Department of Mechanics and Engineering Science, College of Engineering, Peking University, Beijing 100871, China

* Correspondence: yfwang2@pku.edu.cn (Y.F. W); chxhuang@scu.edu.cn (C.X. H)

Figure S1 shows the cross-sectional hardness and martensite content along the depth at different treatment times. And by comparison, it is found that the cross-sectional hardness of S_{10min} sample is significantly different, and the surface martensite content is as high as ~74%. This provides a better preparation for obtaining 3D heterostructure by later annealing. When the treatment time is extended, the hardness and martensite content of the core position will increase, which is not conducive to the structure of the gradient structure. Therefore, the 10 min processing time is selected as the As-SMATed sample here.

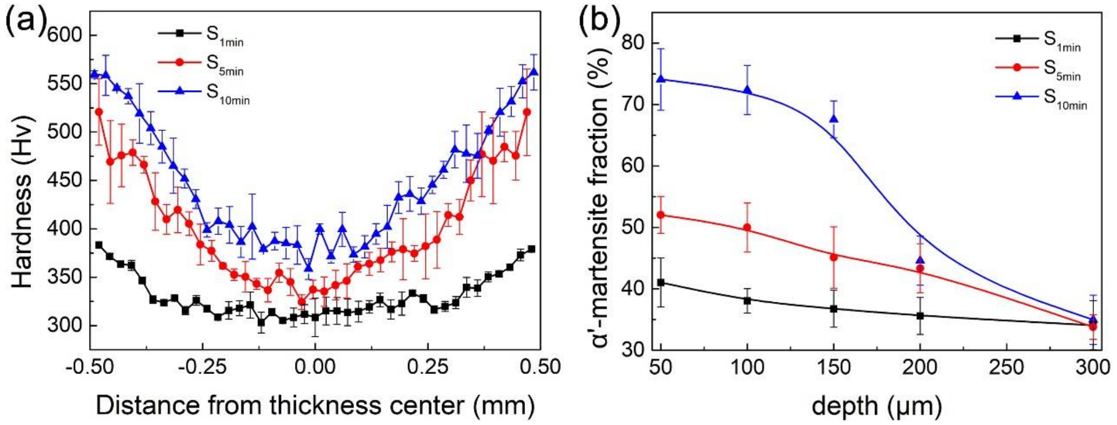

Figure S1. (**a**) Cross-sectional hardness distribution. (**b**) Variation of martensite volume fraction along depth. Three types of gradient plates were symmetrically treated on both sides for 1 min, 5 min and 10 min, which are labeled as S_{1min}, S_{5min} and S_{10min} samples, respectively.

By annealing the as-SMATed samples at different temperatures for 10 s (Figure S2), it is found that the surface hardness of the sample will drop significantly when the temperature rises to 900 °C. This indicates that the martensite recovers and the grains partially recrystallize on the sample surface at 900 °C. This is in line with the 3D heterostructure we want to construct, so 900 °C is chosen as the annealing temperature in the present investigation.

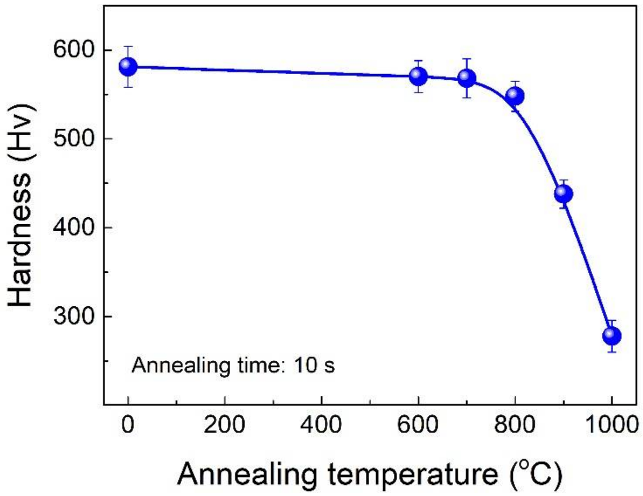

Figure S2. The surface hardness of As-SMATed samples as a function of annealing temperature.

With the extension of the annealing time, the surface hardness of the samples gradually decreases, indicating that the recovery of surface martensite and the number of recrystallized grains are increasing. When the annealing time is above ~25 s, the surface hardness tends to be stable and close to the core. In order to ensure the excellent mechanical properties of the material, the annealing time 5–20 s was selected as the research object.

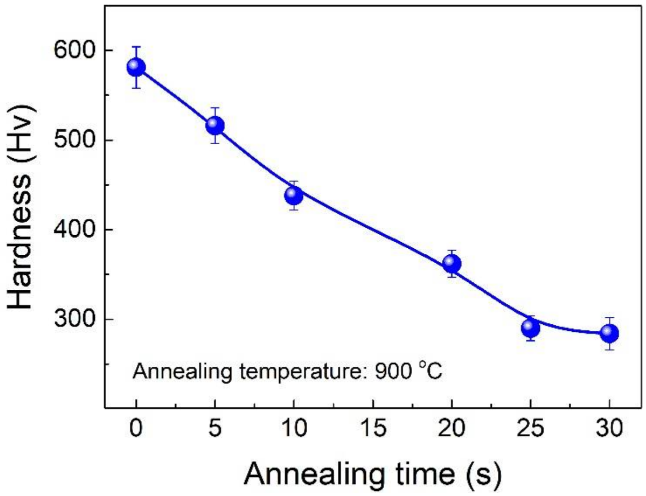

Figure S3. The surface hardness of As-SMATed samples as a function of annealing time.

In Figure S4, austenite is the matrix, twins and α'-martensite are marked with blue and red arrows respectively, and ε-martensite is the green dashed part.

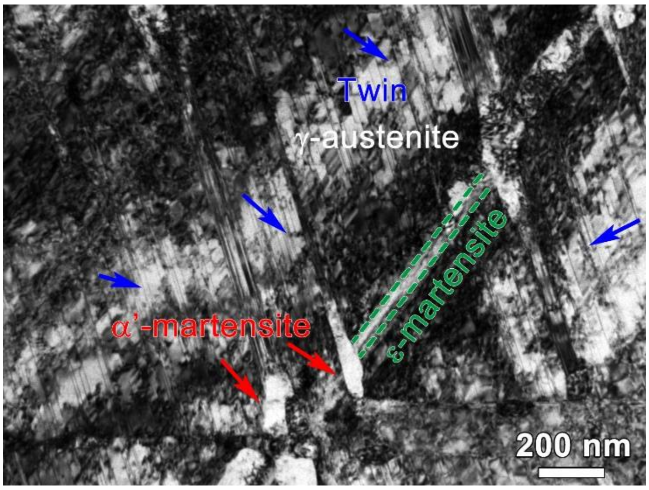

Figure S4. TEM image showing the austenite, twins, ε-martensite and α'-martensite of as-SMATed sample.
